# THE RISK OF MEDICATIONS IMPAIRING ROAD TEST PERFORMANCE AMONG COGNITIVELY INTACT OLDER ADULTS

**DOI:** 10.1093/geroni/igad104.3660

**Published:** 2023-12-21

**Authors:** David Carr, Kebede Beyene, Ganesh Babulal

**Affiliations:** Washington University in St. Louis, St. Louis, Missouri, United States; St. Louis College of Pharmacy, St. Louis, Missouri, United States; Washington University School of Medicine, St. Louis, Missouri, United States

## Abstract

**Importance:**

Older adults are increasingly prescribed medications that have adverse side-effects. Prior studies have found higher risks associated with motor vehicle crashes. Objective: This 10-year study determined whether specific medication classes were associated with performance decline assessed by a standardized road test in a community-sample of healthy, cognitively-normal older adults. Design, Setting, and Participants: This was a prospective cohort study of 198 cognitively-normal older adults ≥ 65 years old with a valid driver’s license who were followed annually, with rolling enrollment. Exposure: Potentially driver impairing medications. Main outcomes and measures: The primary outcome measure was performance (pass or marginal/fail) based on the Washington University Road Test. Multivariable Cox proportional hazards models evaluated the association between PDI medication use and road test performance.

**Results:**

Among the 198 older adults (mean [SD] baseline age, 72.6 [4.6] years; 87 females [43.9%]; and 19 Black participants [9.5%]), 70 participants [35%] received a marginal/fail rating on the road test over an average follow up of 5.70 years. Any antidepressant (HR, 2.81; 95% CI, 1.67- 4.71), SSRI/SNRI (HR, 2.73; 95% CI, 1.56- 4.77), sedatives/hypnotics (HR, 2.70; 95% CI, 1.40- 5.19), or NSAIDs/Acetaminophen (HR, 2.80; 95% CI, 1.34- 5.83) use had at least a 2.5 greater risk of receiving a marginal/fail rating on a road test compared to controls. Conclusion and relevance: In this prospective cohort study, specific medication classes increased the longitudinal risk of failing a road test. Patients should be counseled about perceived driving difficulty and risk when prescribed these medications by clinicians.

